# Implementing the SAFE@home digital platform for blood pressure home monitoring for patients with (a risk of) hypertensive disorders of pregnancy: A barrier and facilitator analysis among obstetric healthcare professionals

**DOI:** 10.1177/20552076251376518

**Published:** 2025-10-23

**Authors:** Shinta L Moes, Martine Depmann, Kvamme Ingelin, Elles In ’T Anker, Jacques Dirken, Leonoor Van Eerden, Arie Franx, Roel De Heus, Sanne J Gordijn, Steven Koenen, Maarten MH Lahr, Titia A Lely, Flip Van Der Made, Lindy Santegoets, Marc Spaanderman, Ewoud Schuit, Mireille N Bekker

**Affiliations:** 1 Department of Obstetrics and Gynecology, 8124 University Medical Centre Utrecht, Utrecht University, Utrecht, The Netherlands; 2 Julius Center for Health Sciences and Primary Care, 168086 University Medical Centre Utrecht, Utrecht University, Utrecht, The Netherlands; 3 Department of Obstetrics and Gynecology, 37226 Bravis Hospital, Bergen op Zoom, The Netherlands; 4 Department of Obstetrics and Gynecology, 10233 Jeroen Bosch Hospital, 's Hertogenbosch, The Netherlands; 5 Department of Obstetrics and Gynecology, 7000 Maasstad Hospital, Rotterdam, The Netherlands; 6 Department of Obstetrics and Gynecology, 6993 Erasmus Medical Center, Rotterdam, The Netherlands; 7 Department of Obstetrics and Gynecology, 6028 St Antonius Hospital, Nieuwegein, The Netherlands; 8 Department of Obstetrics and Gynecology, 10173 University Medical Center Groningen, Groningen, The Netherlands; 9 Department of Obstetrics and Gynecology, 7898 Elisabeth Tweesteden Hospital/Fam, Tilburg, The Netherlands; 10 Department of Epidemiology, 10173 University Medical Center Groningen, Groningen, The Netherlands; 11 Department of Obstetrics and Gynecology, 6992 St Franciscus Gasthuis Hospital, Rotterdam, The Netherlands; 12 Department of Obstetrics and Gynecology, 84744 Reinier de Graaf Hospital, Delft, The Netherlands; 13 Department of Obstetrics and Gynecology, 199236 Maastricht University Medical Center, Maastricht, The Netherlands

**Keywords:** Implementation, digital health, obstetrics, telemonitor, blood pressure, healthcare professional

## Abstract

**Introduction:**

Hypertensive disorders of pregnancy complicate at least 10% of pregnancies, and are associated with fetomaternal morbidity and mortality. Traditional management is resource-intensive. Remote monitoring offers a safe and patient friendly alternative to managing hypertensive disorders. However, scaling telemonitoring requires understanding of implementation facilitators and barriers. This study explores the implementation of SAFE@home, a platform for monitoring blood pressure and symptoms, among obstetric healthcare professionals (OHPs).

**Material and methods:**

An implementation study was conducted across 11 Dutch hospitals from November 2020 to December 2023, evaluating implementation of a home-monitoring platform for pregnant women with a high risk of or with established hypertensive disorders. Implementation outcomes were evaluated using Proctor's taxonomy, Normalization Measurement Development and Measurement Instrument for Determinants of Innovations questionnaires, platform, and electronic health records data. OHPs received questionnaires at the start of implementation (*T* = 1) and between 9 and 18 months (*T* = 2). Median and proportion of scores were analyzed.

**Results:**

Of 133 OHPs, 83 (62.4%) and 101 (75.9%) responded to *T* = 1 and *T* = 2, respectively. SAFE@home was well-received, with a median score of 4/5 on questionnaires at both time points. Adoption was 100%, and OHPs were receptive to using the platform (95% at *T* = 1; 92% at *T* = 2). Confidence in using SAFE@home was 48% (*T* = 1) and 85% (*T* = 2); 86% believed it improved outcomes and 82% found work integration easy. Overall, 91% anticipated SAFE@home to become routine. Barriers included time constraints (46%) and uncertainty about usage (20%).

**Conclusions:**

SAFE@home was successfully implemented, with high acceptance and sustainability potential. While time constraints and initial uncertainty were barriers, most OHPs believed telemonitoring could improve care for high-risk pregnancies.

## Introduction

Hypertensive disorders of pregnancy (HDPs) are a prevalent complication, affecting at least 10% of pregnancies globally, and present a significant clinical challenge to obstetric healthcare. These disorders rank among the leading causes of maternal and fetal or neonatal morbidity and mortality, emphasizing the urgent need to innovate prevention and management strategies.^1–3^

The traditional model of frequent outpatient visits and in-hospital monitoring of blood pressure (BP) and HDP-related symptoms is resource-intensive.^4^ Recent advancements in digital health technology, particularly remote monitoring, offer a transformative approach to this challenge.^5^

Digital health innovations, including home monitoring of BP in pregnancy, have demonstrated safety and increasing feasibility, providing ample evidence to support a shift toward more proactive and personalized healthcare strategies. The blood pressure monitoring in high risk pregnancy to improve the detection and monitoring of hypertension (BUMP) 1 trial in the UK showed that self-monitoring of BP in pregnancy was not only feasible, but also led to earlier detection of hypertension, and the BUMP 2 trial demonstrated safety and high acceptance among women with chronic or gestational hypertension.^6,7^ Similarly, Lanssens et al. reported reduced in-person visits and high-patient satisfaction in a Belgian cohort using a remote-monitoring program for gestational hypertensive disorders.^8^ These studies emphasize the clinical value and acceptability of self-monitoring, but do not address the implementation process from the perspective of healthcare professionals. In recent years the SAFE@home platform, a digital platform for monitoring BP- and HDP-related symptoms in women with high risk of or with established HDP was developed in the Netherlands.^9,10^ This platform facilitated timely interventions while reducing the necessity for frequent in-person visits at the hospital. This approach not only enhanced patient convenience but also optimized healthcare resource allocation. Gaining insight into the facilitators and barriers of implementation is essential for achieving sustainable innovation on a larger scale. Currently however, only small-pilot studies have explored these factors.

In this prospective cohort study, we aim to systematically explore the barriers and facilitators of the implementation of the SAFE@home platform, specifically from the perspective of obstetric healthcare professionals (OHPs). Through a comprehensive analysis of the implementation landscape, our goal is to identify key factors that influence the adoption of the SAFE@home platform, offering evidence-based recommendations to enhance digital healthcare and promote sustained uptake. Our findings will offer insights to clinicians, policymakers, and digital health developers leading to improved implementation of healthcare innovations beyond the field of obstetric care.

## Materials and methods

### Study design and population

A quantitative study evaluating implementation outcomes of telemonitoring among OHPs was conducted within the SAFE@home II study. The SAFE@home II study was a multicenter non-randomized non-inferiority before-after study with a retrospective control group conducted in 11 hospitals across the Netherlands from November 2020 until December 2023. To assess the safety and clinical impact of a digital home-monitoring platform (SAFE@home) as a replacement for usual care, women with a singleton pregnancy who had a risk of HDP or with established HDP, that is, chronic hypertension, history of preeclampsia, maternal cardiac disease, maternal kidney disease, or pregnancy-induced hypertension before 34 weeks were included. Eligible women were ≥18 years old and had access to a smartphone or tablet with internet connection and sufficient knowledge in Dutch or English. A body mass index (BMI) > 35 kg/m^2^ or an arm circumference <22 or >42 cm (due to technical requirements of the BP cuff) were exclusion criteria. The prospective cohort used the SAFE@home platform during a fixed time period of 18 months in each participating center. During these 18 months, women were offered home monitoring of BP and symptoms in combination with a hybrid care path containing a reduced number of scheduled antenatal visits.

Since the goal of the implementation study was to evaluate the experiences and perceptions of all OHPs directly involved in the SAFE@home platform, we aimed to survey the entire population of eligible professionals across the 11 participating hospitals. As such, no formal sample-size calculation was performed. Instead, all nurses, hospital-based midwives, residents, and gynecologists who actively worked with or supervised the use of the platform were invited to complete the questionnaires. This approach was chosen to ensure a complete representation of the perspectives relevant to implementation.

### OHP roles during implementation of SAFE@home

All participating OHPs completed a standardized training program in use of the telemonitoring platform, learning to access incoming BP and symptom data either through the local electronic health record or via a secure web portal, according to each hospital's setup. Nurses and midwives were primarily responsible for instructing patients on how to use the SAFE@home platform, including guidance on taking accurate BP measurements, ability to use the symptom questionnaire, and submitting data through the application. Nurses, midwives, and physicians had day-to-day responsibility for reviewing all patient-submitted measurements above the alarming threshold against a predefined protocol. Any worrying values or questions raised during review were escalated to and supervised by an attending gynecologist or gynecologist in training. At their discretion or per supervisory advice, OHPs could initiate telephone outreach or arrange in-person visits. The system also supported one-way messaging from OHPs to patients, enabling brief instructions or reassurance to be sent directly through the platform.

### Implementation definitions and outcomes

To evaluate the barriers and successes of the implementation of a digital innovation in the field of obstetrics, we used Proctor's taxonomy for implementation outcomes to assess implementation success among OHPs.^11–13^ Proctor's taxonomy outlines the results achieved at various stages of implementation. At the same time the actions and behaviors of OHPs were examined as they work to integrate a new approach into their daily practice.

We predefined the indicators per domain of implementation for OHPs. Evaluation of the different indicators among OHPs was performed using the Measurement Instrument for Determinants of Innovations (MIDI) and administrative datasets.^14^ Furthermore, we evaluated the process of this implementation among OHPs using the validated Normalization Measurement Development (NoMAD) instrument.^15–17^ The implementation outcomes, definitions, and method for assessment in this study's context are summarized in Table 1.

**Table 1.** Proctor's taxonomy of implementation outcomes.^a^

Outcome	Definition	Indicator	Assessment method
Acceptability	Perception among OHPs that SAFE@home is agreeable or satisfactory for the OHP and to the patient that uses the platform	Perceived acceptability of SAFE@home regarding effectiveness and decrease of workload	NoMAD C1, C3, C4, RM1–3, MIDI I1, I2, I4, U8, U17, U18
Adoption	Initial decision of (a) a hospital to implement SAFE@home, and (b) of OHP to offer SAFE@home to their patients	The number of hospitals that started with SAFE@home divided by the total number of hospitals that was approached (observation)	NoMAD CP3 + administrative data
Appropriateness	(a) Perceived fit, relevance, or compatibility of SAFE@home for OHP, and (b) their goal to personalize care and improve quality	The number of OHPs who appreciated SAFE@home the impact it had on personalizing and quality of care, divided by total number of OHPs who responded to the questionnaire	NoMAD GN1, C2, CP2, MIDI I7, U9, U11, U12 Extra E3
Feasibility	Extent to which SAFE@home can be successfully used or carried out within the hospital	Proportion of OHP that can integrate SAFE@home into their work and feel they have enough time and resources to implement the SAFE@home	NoMAD GN2, CP1, CA1–7, RM5, MIDI U13, U16, U19, O21–24, O26, O27
Fidelity	Extent to which patient adhered to the predefined measurement schedule	Number of measurements performed, divided by total measurements possible	Data analysis of the data collected through EHR and sample analysis
	Extent to which number of antenatal outpatient visits was in line with the prescribed care path	Number of pregnant women that received the correct care path, divided by the total number of women who were included	Sample analysis (of 1 academic and 1 general hospital) in EHR
	Extent to which right patients were included for SAFE@home monitoring	Included patients, divided by enrolled patients	Analysis of medical records
Penetration	Integration of SAFE@home in hospitals	Penetration rate of SAFE@home among intended users within the organization	MIDI U14, U15, U18
Sustainability	Intention to use SAFE@home after study period	The number of hospitals with the intention to continue use of SAFE@home after the study period, divided by the total number of hospitals that participated in the study	NoMAD GN3, CP4, RM4, MIDI I6, O20, O28 Extra E1, E2, E4

OHP: obstetric healthcare professional; NoMAD: Normalization Measurement Development; MIDI: Measurement Instrument for Determinants of Innovations; EHR: Electronic Health Record.

a Outcome column shows the different Proctor domains. Definition column explains our definition of the Proctor domain with regards to the SAFE@home platform implementation. The indicator column shows how we measure the defined outcome and the assessment column depicts the actual questions and methods used to collect the necessary data for the indicators.

### Data collection

OHPs of four academic hospitals and seven general hospitals received the NoMAD and MIDI questionnaires at two predefined time points. The first time point (*T* = 1) was scheduled at 4 weeks after the start of SAFE@home implementation at each hospital, allowing for initial exposure to the platform. The second time point (*T* = 2) was planned to capture longer-term experiences and was set between 9 and 18 months since implementation. This time frame was chosen to account for variability in hospital-specific rollout timelines and the dynamic composition of OHP teams, while also ensuring sufficient response rates. The *T* = 2 measurement thus captures OHP perspectives reflecting experienced interaction with the platform.

Participation in the study was voluntary, and informed consent was obtained through an implied consent procedure, appropriate for minimal-risk survey research. All OHPs received an invitation email explaining the purpose of the study, the anonymous and non-traceable nature of their responses, and that results would be analyzed at the group level only. All questionnaire items are listed in Tables S1, S2, and S3. Collected demographics included age, profession (categorized as gynecologists, midwives, residents, and nurses), and years of experience in the current hospital and the obstetric field. Furthermore, data was retrieved from electronic records of the participating hospitals to complete analyses for the remaining Proctor domains.

### Statistical analysis

The NoMAD and MIDI questionnaires were scored on a 5-point Likert scale from 0 (strongly disagree) to 5 (strongly agree). Items on which ≥20% of patients or OHPs responded “disagree” or “strongly disagree” were considered barriers of implementation and items on which ≥80% responded “agree” or “strongly agree” were considered facilitators of implementation, these cutoff points were used following the methods of previous research.^18,19^ The internal consistency, to test if items are measuring the same concept and are answered in the same direction, was evaluated using Cronbach's alpha. The alpha values were interpreted as follows: *α* < 0.50 “unacceptable,” *α* 0.50 to 0.59 “poor,” *α* 0.60 to 0.69 “questionable,” *α* 0.70 to 0.79 “acceptable,” *α* 0.80 to 0.89 “good,” and *α* ≥ 0.90 “excellent.” Median (interquartile range (IQR)) scores were calculated for all items of the questionnaire. In addition, for the following professional subgroups of OHPs: nurses, midwives, and gynecologists, descriptive subgroup analyses were conducted to explore whether notable differences or similarities in distribution of answers between professional cadres existed. Data analyses were performed in R version 4.2.3 and SPSS statistics version 29.0.1 (SPSS Inc., Chicago, IL).

## Results

A total of 83 (62.4%) and 101 (75.9%) out of 133 OHPs responded to the questionnaires sent at *T* = 1 and *T* = 2, respectively. Of these respondents, 41 (30.8%) responded to both questionnaires, 42 (31.6%) only responded at *T* = 1, and 61 (45.9%) only at *T* = 2. OHP characteristics are given in Table 2. Response rates per center are given in Appendix Table S4.

**Table 2.** Characteristics of obstetric healthcare professionals.

	*T* = 1, *N* = 83	*T* = 2, *N* = 101
Gender of OHP, *n* (%)		
Male	9 (10.8%)	18 (17.8%)
Female	74 (89.2%)	83 (82.2%)
Age, mean (SD)	45 (11)	43 (11)
Center of employment OHP, *n* (%)		
Site 1	15 (18.1%)	12 (11.9%)
Site 2	8 (9.6%)	8 (7.9%)
Site 3	5 (6.0%)	9 (8.9%)
Site 4	1 (1.2%)	2 (2.0%)
Site 5	12 (14.5%)	21 (20.8%)
Site 6	16 (19.3%)	6 (5.9%)
Site 7	9 (10.8%)	17 (16.8%)
Site 8	6 (7.2%)	19 (18.8%)
Site 9	4 (4.8%)	4 (4.0%)
Site 10	5 (6.0%)	2 (1.6%)
Site 11	2 (2.4%)	1 (0.8%)
Profession, *n* (%)		
Nurse	25 (30.1%)	22 (21.8%)
(Clinical) obstetrician	18 (21.7%)	16 (15.8%)
Gynecologist	23 (27.7%)	31 (30.7%)
Doctor not in training	4 (4.8%)	11 (10.9%)
Physician assistant	2 (2.4%)	5 (5.0%)
Doctor in training	6 (7.2%)	5 (5.0%)
Other	5 (6.0%)	11 (10.9%)
Years of experience in obstetrics, mean (SD)	19 (11)	16 (11)
Years working in current hospital, mean (SD)	13 (11)	13 (11)
Role in working with SAFE@home, *n* (%)		
1. Actively working with SAFE@home	47 (56.6%)	82 (81.1%)
2. Supervising work of 1	26 (31.3%)	12 (11.9%)
3. Otherwise	10 (12.0%)	8 (7.9%)

*n*: number; OHP: obstetric healthcare professional; SD: standard deviation.

### Implementation outcomes

**Figure 1.**
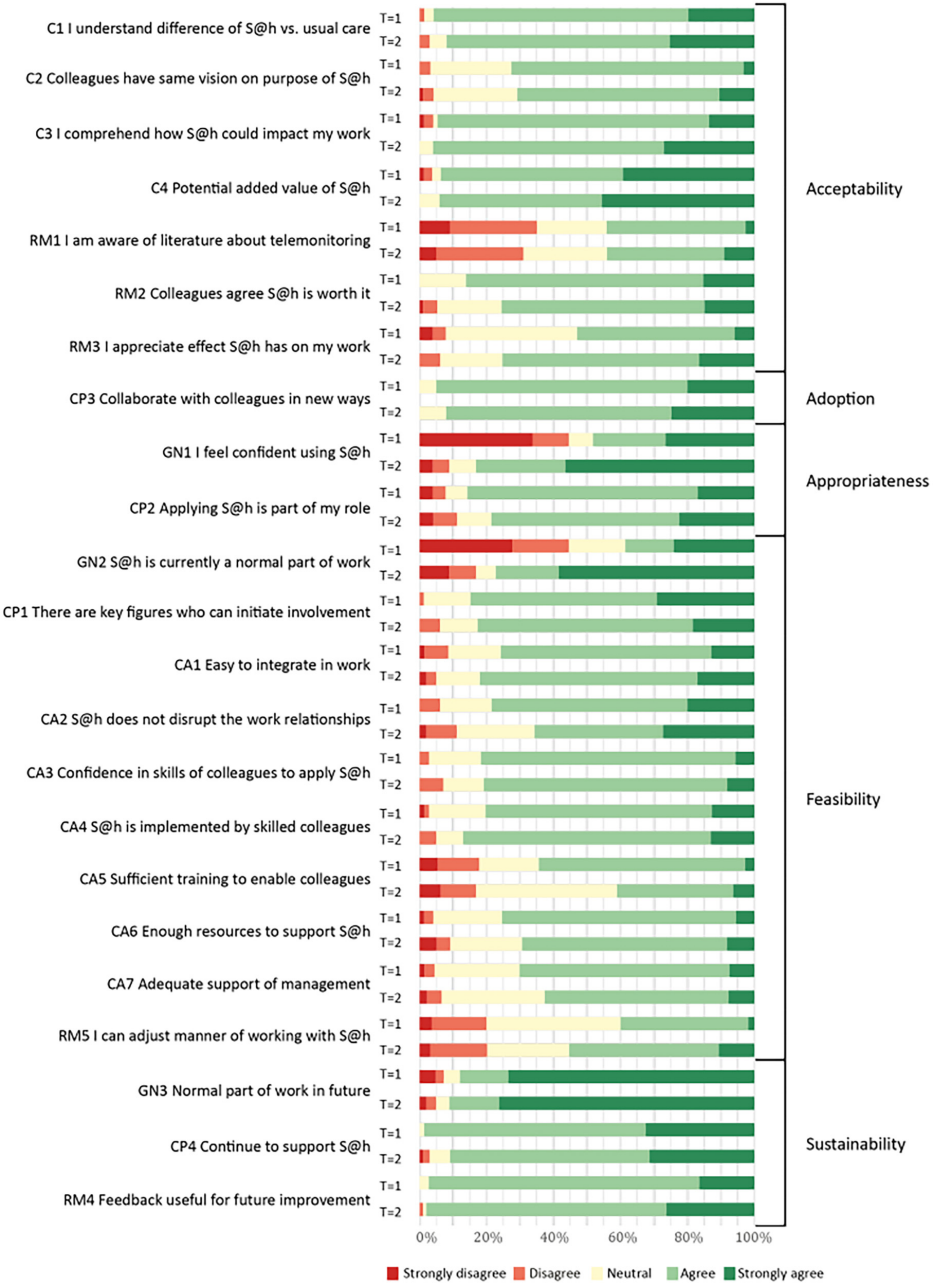
Distribution of answers from OHPs to NoMAD questionnaire at *T* = 1 and *T* = 2. S@h: SAFE@home; OHP: obstetric healthcare professional; NoMAD: Normalization Measurement Development.

**Figure 2.**
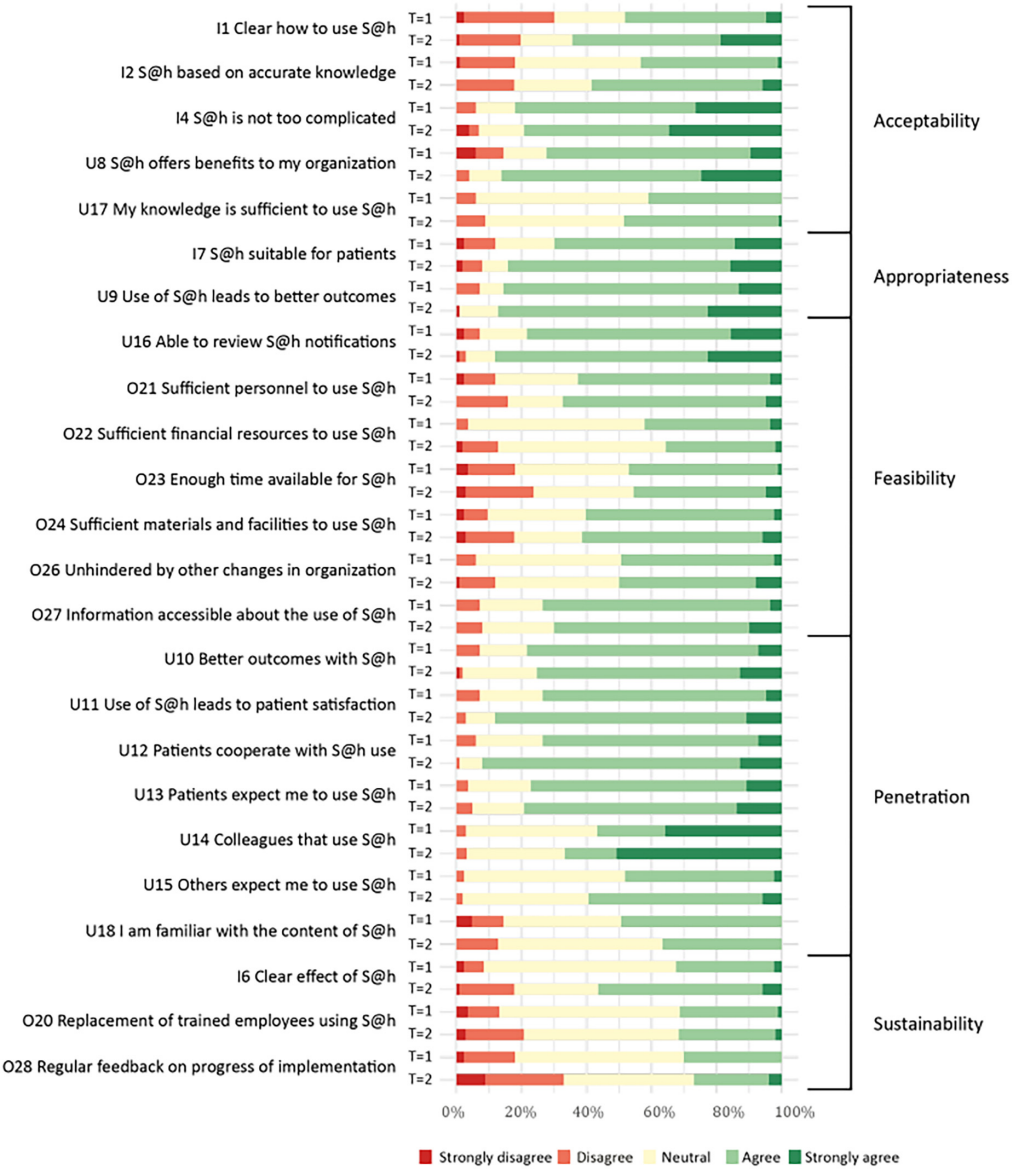
Distribution of answers from OHPs to MIDI questionnaire at *T* = 1 and *T* = 2. S@h, SAFE@home; OHP: obstetric healthcare professional; MIDI: Measurement Instrument for Determinants of Innovations.

With an overall score of 4 out of 5 (IQR 3–4) for the MIDI and NoMAD questionnaires at the start of the implementation process, the OHPs were predominantly positive about the SAFE@home platform. In the second half of the implementation period the median overall score remained a stable 4 out of 5 (IQR 3–4). The overall internal consistency (*α*) of the questionnaires was good, ranging from 0.80 to 0.86 at *T* = 1 and from 0.85 to 0.89 at *T* = 2 (Table S5 in the Appendix). Proportion of all answers by respondents of the NoMAD and MIDI questionnaire at *T* = 1 and *T* = 2 can be found in Figures 1 and 2, respectively.

### Adoption

The institutional level adoption rate for SAFE@home was 100%, meaning all of the hospitals that initially intended to implement the platform successfully integrated it into their clinical care processes. At the individual level of the OHP, 95% (*n* = 76) of them were receptive to work with their colleagues in new ways using SAFE@home in the early implementation phase (*T* = 1) and in 92% (*n* = 93) this openness continued to be prevalent in the second half of the implementation period (CP3).

### Acceptability

#### Facilitators

The majority of OHPs (*n* = 74; 94% and *n* = 95; 94%) saw the potential added value of SAFE@home, at both *T* = 1 and *T* = 2, respectively (C4). Regarding benefits for the organization, 83% (*n* = 84) of OHPs believed SAFE@home would offer better patient care at *T* = 2 (U8a). Also at *T* = 2, 72% of OHPs appreciated how SAFE@home affected their work, compared to 53% at *T* = 1 (RM3).

#### Barriers

At *T* = 1, 30% of OHPs (*n* = 25) reported a lack of clarity regarding the activities required when using SAFE@home, indicating an initial barrier to implementation. At *T* = 2 however, the lack of clarity was as low as 20% (*n* = 20), with the majority of OHPs (64%, *n* = 65) clearly understanding the necessary activities, reflecting an improvement as the intervention progressed (I1). Another barrier at the initiation of the study was the unawareness of literature on telemonitoring, which was 41%, *n* = 34 at *T* = 1 and 31%, *n* = 31 at *T* = 2 (RM1).

### Appropriateness

#### Facilitators

At *T* = 1, 80% (*n* = 66) of OHPs believed that using SAFE@home was a part of their role. This continued to be the case for 78% (*n* = 77) at *T* = 2 (CP2). The proportion of OHPs that felt confident using SAFE@home was 48% (*n* = 40) at *T* = 1, and was as high as 85% (*n* = 84) at *T* = 2 (GN1). The amount of OHPs that found SAFE@home suitable for their patients was 70% (*n* = 58) at *T* = 1, and 84% (*n* = 85) at *T* = 2 (I7). A majority of OHPs (86%; *n* = 71) also expected that SAFE@home could result in better outcomes for their patients. At *T* = 2 only 4% (*n* = 4) of OHPs believed this platform would not benefit patient outcomes (U9). Regarding the frequency of BP measurements, 77.1% (*n* = 64) of OHPs believed it to be appropriate at *T* = 1. At *T* = 2, 84% (*n* = 84) found the frequency appropriate, with 14% (*n* = 14) indicating that measurements were performed too often and 2% (*n* = 2) too few (E3).

#### Barriers

At the start of the implementation of SAFE@home (*T* = 1), 49% (*n* = 41) of OHPs did not feel confident using the platform.

### Feasibility

#### Facilitators

At *T* = 2, 82% (*n* = 82) of OHPs reported that integrating SAFE@home into their work was easy (CA1). Moreover, 67% (*n* = 68) felt that enough resources were available to support the platform at *T* = 2 (CA6). Regarding the ability to review the notifications the program generated, 78% (*n* = 65) indicated they were able to at *T* = 1, and 88% of OHPs were able to (*n* = 89) at *T* = 2 (U16). SAFE@home being implemented by skilled colleagues was a facilitator at *T* = 2 with 86% (*n* = 87) of OHPs confirming this (CA4).

#### Barriers

At *T* = 2, with 46% (*n* = 46) of OHPs reporting to have insufficient time to integrate SAFE@home into their daily routine at work (O23), this was identified as a barrier.

Other resources were neither seen as a barrier nor a facilitator to implementation, with 50% to 80% indicating sufficient staff capacity (O21), financial resources (O22), materials and facilities (O24) to use the SAFE@home platform at *T* = 1 as well as *T* = 2. At *T* = 1, 57% reported training to enable colleagues to implement SAFE@home as adequate. At *T* = 2, 39% felt training was adequate (CA5).

### Fidelity

Median adherence to the measurement protocol was 86% (IQR 75–95%) in the overall study cohort. The predefined number of outpatient visits to be scheduled during SAFE@home was 11. The median number of outpatient visits in the SAFE@home cohort was 11 (IQR 8–13). Furthermore, from the sample analysis of one academic and one general hospital, the median number of outpatient visits were 10 and 11, respectively. Out of 756 enrolled patients, 88 (11.6%) were excluded for failing to meet the predefined inclusion criteria. These exclusions were due to patients either lacking a risk factor of HDP or established HDP at the time of enrollment, or having a gestational age beyond the specified 12–34 week range at enrollment. Furthermore, 62 (8.2%) of the patients met an exclusion criterion, with the majority having a BMI above 35 kg/m^2^.

### Penetration

At *T* = 1, almost half of OHPs (47%, *n* = 39) were influenced by the perceived expectations or opinions of their colleagues, managers, or patients to use the platform. By *T* = 2, this was 45% (*n* = 74) (U15), with 49% of OHPs caring about the opinions of colleagues and 30% about those of managers (30%). Additionally, 65% (*n* = 65) of OHPs believed that the colleagues for whom SAFE@home was intended were indeed using it at *T* = 2 (U14).

#### Facilitators

With 82% (*n* = 83) of OHPs that did care about the opinions of patients, this was a facilitator for penetration. A majority of OHPs believed that patients would cooperate (*T* = 1: *n* = 61; 73%, *T* = 2: *n* = 93; 92%) (U12) and be satisfied (*T* = 1: *n* = 61; 73%, *T* = 2: *n* = 89; 88%) (U11) when using SAFE@home.

OHPs believed that the patients would expect them to use the platform (*T* = 1: *n* = 64; 77%, *T* = 2: *n* = 80; 79%) (U13).

#### Barriers

There were no barriers identified to the penetration of SAFE@home implementation.

### Sustainability

At the last part of the implementation period (*T* = 2) 77% (*n* = 78) of OHPs would continue to support the use of SAFE@home (E1).

#### Facilitator

Among the OHPs, 91% (*n* = 92) believed it would become a normal part of their work in the future (GN3). Of the participating hospitals, 10 out of 11 (90.1%) expressed the intention to continue home monitoring of BP and health complaints.

#### Barriers

An identified barrier was a lack of regular feedback on the progress of the SAFE@home implementation (O28), as indicated by 34% disagreement (*n* = 43) at *T* = 2.

### Perceptions among nurses, midwives, and gynecologists

No substantial differences were observed in the distribution of answers between nurses, midwives, and gynecologists. Due to small numbers formal subgroup analysis for all cadres was not feasible. Detailed item-level distributions of nurses, midwives and gynecologists are provided in Appendix Figures S1 to S3.

## Discussion

### Principal results

In this prospective cohort study, we systematically explored the barriers and facilitators involved in the implementation of the SAFE@home platform. Our findings demonstrated its successful adoption. Furthermore, with 83 and 101 OHPs responding to the NoMAD and MIDI questionnaires at two predefined time points, we identified key factors influencing the effective implementation of digital healthcare in routine clinical practice. The most important facilitators included confidence in using the platform, a shared vision among colleagues regarding SAFE@home, the expectation that its use would lead to improved patient outcomes, collaboration, and a clear understanding of how the intervention could impact future work. The most important barriers were initial complexity, lack of time and resources, limited feedback on the implementation progress, and insufficient flexibility in the manner of working with SAFE@home. The insights from our study have potential applications beyond the field of pregnancy care.

### Strengths and limitations

Our study has several strengths. First, it provides a comprehensive evaluation of the implementation of the SAFE@home telemonitoring platform using Proctor's taxonomy and validated measurement tools. This structured approach ensures a robust and evidence-based assessment of facilitators and barriers to digital health adoption in obstetric care. Second, the study is a large, multicenter evaluation conducted across 11 hospitals with high response rates, offering real-world, generalizable insights into the integration of telemonitoring among OHPs. However, our study has some limitations that need to be addressed. First, the use of self-reported questionnaires might have introduced response bias, as OHPs with strong opinions or feelings might have been more likely to respond while those with more moderate opinions might be less inclined to participate. However, the good response rates in this study suggest a strong level of engagement. When researching implementation, other stakeholders involved in the implementation process such as information and communication technology or medical technology departments could have been questioned. This was not done in our study. However, the OHPs together with the patients are the most important stakeholders for this innovation to work sustainably. For future research, incorporating other stakeholders may give a more comprehensive perspective on the implementation process.

Furthermore, our study included various professionals with different tasks and roles in working with SAFE@home, which may have introduced variability in responses due to difference in the level of experience and perspectives. On the other hand, by including this variety of professionals, the overall score represents real life practice and experiences throughout the centers.

Lastly, the respondent groups at *T* = 1 and *T* = 2 differed significantly, with only one-third overlap between the two groups. This limited overlap means that the present study cannot be fully considered longitudinal, making it challenging to draw definitive conclusions about decreases or increases of factors influencing implementation over time.

### Comparison with prior work

A previous pilot study demonstrated feasibility and satisfaction among patients using the SAFE@home platform.^9,20^ The present study builds on this previous knowledge by adding the OHP perspective on digital innovation on a much larger scale. A review of Whitelaw et al. investigating the uptake of digital health innovations in cardiovascular care included 29 studies, of which four studies reported clinician-level facilitators.^21^ The most frequent facilitators identified included approval and organizational support from cardiology departments and/or hospitals, technologies that improved efficiency and technologies that clinicians perceived as useful. In line with this review, we found that organizational support and perceived usefulness of technology were important facilitators of implementation. However, in our study the efficiency was a barrier in terms of time constraints and inadequate training throughout the implementation period.

A review on digital health competencies in healthcare professionals that included 26 studies,^22^ identified the importance of a positive attitude and the ability to emotionally adapt for successful adoption of digital technologies. In our study, this was reflected by the perceived usefulness of SAFE@home and confidence in use of the platform at the end of the implementation period. The review also revealed methodological weaknesses, like the use of non-validated tools in the majority of the included studies, which raised concerns about the reliability of the findings. Our study addressed the limitations raised in this review by using validated questionnaires, which have already been tested for reliability and accuracy, providing more trustworthy findings.^16–19^ A review by Gijsbers et al., that identified the enablers and barriers for upscaling of telemonitoring, confirmed the challenges regarding sufficiency of resources and the need for OHP training. This study also paralleled the importance of institutional support to achieve broader adoption.^23^ A qualitative study in Ghana also researched barriers and facilitators of home BP monitoring during pregnancy, showing the same perspective of the potential of telemonitoring to improve patient outcomes.^24^ However, this study was primary focused on the equipment costs and communication infrastructure in a lower-resource setting. SAFE@home on the other hand was studied in a high-resource setting with a focus on work integration and managing time constraints, rather than material barriers. Our study builds upon prior research by reaffirming the significance of organizational support, perceived usefulness, and OHP competencies in the adoption of digital health innovations. In particular, we observed differences in timing and development across the participating centers during the implementation phase, which may be attributed to local cultural variations and the existence of a learning curve. For instance, adherence to the care pathway with fewer visits proved challenging for OHPs in situations where uncertainty about a patient's health status arose.

### Recommendations

The implementation of SAFE@home provided several key lessons for successful adoption of digital health interventions. Organizational support is essential, as early institutional commitment can facilitate widespread adoption. Continuous training throughout the implementation process is critical to maintain clarity, avoid confusion about required activities, and prevent a decline in satisfaction with digital health over time. Additionally, it is vital to find ways to integrate the use of digital platforms into daily routines of OHPs effectively and to encourage their use as consistent exposure might help build confidence. Peer support, provided by the involvement of skilled colleagues, plays a key role in facilitating the transition. OHPs should be actively involved in evaluations throughout the implementation period to monitor progress and adherence at different phases. This approach ensures alignment with local practices and enhances trust in the platform, ultimately improving the acceptability of digital health and the fidelity of the reduced visits care path. Finally, supporting the belief in the platform's ability to improve patient care could be essential for long-term sustainability, as it establishes the importance of aligning new technologies with perceived clinical benefits.

## Conclusions

This study investigated the implementation barriers and facilitators of the SAFE@home platform, providing telemonitoring for high-risk pregnancies, among OHPs. We demonstrated high adoption and sustainability across the 11 participating hospitals, with strong acceptance and positive perceptions from OHPs. Despite barriers such as time constraints and initial uncertainty SAFE@home use, most OHPs believed in the potential of telemonitoring to improve care for high-risk pregnancies. Ultimately, these findings support the notion among OHPs that telemonitoring is not just a solution for the future, but a vital part of enhancing healthcare today.

## Supplemental Material

sj-docx-1-dhj-10.1177_20552076251376518 - Supplemental material for Implementing the SAFE@home digital platform for blood pressure home monitoring for patients with (a risk of) hypertensive disorders of pregnancy: A barrier and facilitator analysis among obstetric healthcare professionals Supplemental material, sj-docx-1-dhj-10.1177_20552076251376518 for Implementing the SAFE@home digital platform for blood pressure home monitoring for patients with (a risk of) hypertensive disorders of pregnancy: A barrier and facilitator analysis among obstetric healthcare professionals by Shinta L Moes, Martine Depmann, Kvamme Ingelin, Elles In ’T Anker, Jacques Dirken, Leonoor Van Eerden, Arie Franx, Roel De Heus, Sanne J Gordijn, Steven Koenen, Maarten MH Lahr, Titia A Lely, Flip Van Der Made, Lindy Santegoets, Marc Spaanderman, Ewoud Schuit and Mireille N Bekker in DIGITAL HEALTH

## Abbreviations

IQR: interquartile range
MIDI: Measurement Instrument for Determinants of Innovations
NoMAD: Normalization Measurement Development
OHP: obstetric healthcare professional
S@h: SAFE@home
