# Supplementary material for: Implementing the SAFE@home digital platform for blood pressure home monitoring for patients with (a risk of) hypertensive disorders of pregnancy: A barrier and facilitator analysis among obstetric healthcare professionals
Source: Digit Health. 2025 Oct 23;11:20552076251376518. doi: 10.1177/20552076251376518 (PMC12559661; doi:10.1177/20552076251376518)

Appendix

Table S1. Items of NoMAD questionnaire

| **#** | **Item** |
| --- | --- |
| 1 | I understand how SAFE@home differs from the usual way of working |
| 2 | Colleagues in this organization share the same view about the purpose of SAFE@home |
| 3 | I comprehend how SAFE@home can impact the nature of my own work |
| 4 | I see the potential added value of SAFE@home for my work |
| 5 | I can easily integrate SAFE@home into my current work |
| 6 | SAFE@home disrupts the work relationships within the organization |
| 7 | I have confidence in the skills of colleagues to apply SAFE@home. |
| 8 | SAFE@home is implemented by colleagues who are proficient in it. |
| 9 | Sufficient training is provided to enable colleagues to implement SAFE@home. |
| 10 | There are enough resources available to support SAFE@home. |
| 11 | Management adequately supports SAFE@home. |
| 12 | There are key figures in the organization who can implement and involve others in SAFE@home |
| 13 | I believe that applying SAFE@home is a legitimate part of my role in the organization |
| 14 | I am open to collaborating with colleagues in new ways when using SAFE@home |
| 15 | I will continue to support the use of SAFE@home |
| 16 | Do you feel confident when using SAFE@home |
| 17 | Do you feel that SAFE@home is currently a normal part of your work |
| 18 | Do you think SAFE@home will become a normal part of your work in the future |
| 19 | I am aware of the (professional) literature regarding the effects of telemonitoring. |
| 20 | Colleagues agree that SAFE@home is worth it. |
| 21 | I appreciate the effects that SAFE@home has had on my work. |
| 22 | Feedback regarding SAFE@home can be used to improve it in the future. |
| 23 | I can adjust how I work with SAFE@home. |

Table S2. Items of MIDI questionnaire

| **#** | **Item** |
| --- | --- |
| 1 | It is clear which activities I need to perform and in what sequence when using SAFE@home. |
| 2 | SAFE@home is based on factually accurate knowledge. |
| 3 | SAFE@home is too complicated for me to use. |
| 4 | I clearly observe the effects of using SAFE@home. |
| 5 | I find SAFE@home suitable for my patients. |
| 6 | SAFE@home offers my organization: better patient care/quieter consultation hours/less emergency consultations/increased medicalization |
| 7 | I think it is important using SAFE@home achieves less outpatient visits with / higher patient satisfaction / better blood pressure surveillance/more autonomy for the patient / less admissions due to hypertension |
| 8 | Using SAFE@home I expect less outpatient visits with / higher patient satisfaction / better blood pressure surveillance/more autonomy for the patient / less admissions due to hypertension |
| 9 | Patients will generally be satisfied if I use SAFE@home. |
| 10 | Patients will generally cooperate if I use SAFE@home. |
| 11 | Patients expect me to use SAFE@home |
| 12 | How many of your colleagues in your organization, for whom SAFE@home is intended, actually use SAFE@home? |
| 13 | Fellow healthcare providers / managers / patients expect me to use SAFE@home |
| 14 | If you wanted to, do you think you would be able to review (and process) the notifications from SAFE@home? |
| 15 | I have sufficient knowledge to use SAFE@home. |
| 16 | To what extent are you familiar with the content of SAFE@home? |
| 17 | Are formal agreements regarding the use of SAFE@home established by management in your organization (in policy plans, work plans, instructions, etc.)? |
| 18 | In my organization, measures have been taken to ensure that employees who use SAFE@home and leave the organization are promptly replaced by (new) employees who are adequately trained in the innovation. |
| 19 | There is sufficient personnel in our organization to use SAFE@home as intended. |
| 20 | There are sufficient financial resources available to use SAFE@home as intended. |
| 21 | Our organization provides me with enough time to integrate SAFE@home into my daily work. |
| 22 | Our organization provides me with sufficient materials and facilities to use SAFE@home as intended. |
| 23 | When working with SAFE@home, to what extent do you feel hindered by other changes in the organization? (such as reorganization, merger, budget cuts, staff turnover, other innovations) |
| 24 | In my organization, regular feedback is provided on the progress of the implementation of SAFE@home. |

Table S3. Items of Extra questionnaire

| **#** | **Item** |
| --- | --- |
| 1 | I would like to continue using the digital platform SAFE@home (the Luscii app in combination with the automatic blood pressure monitor) in the future. |
| 2 | I would like to continue using the SAFE@home care pathway in the future. |
| 3 | I find measuring daily on weekdays a) a good frequency, b) too often, c) too little. |
| 4 | Do you have any recommendations or comments regarding the use of SAFE@home in the future? |

|  | **T=1, N= 83** | **T=2, N=101** |
| --- | --- | --- |
| **Center of employment OHP, n (%)** | | |
| Site 1 | 15 / 16 (93.8%) | 12 / 16 (75.0%) |
| Site 2 | 8 / 10 (80.0%) | 8 / 10 (80.0%) |
| Site 3 | 5 / 10 (50.0%) | 9 / 10 (90.0%) |
| Site 4 | 1 / 2 (50.0%) | 2 / 2 (100.0%) |
| Site 5 | 12 / 22 (54.5%) | 21 / 22 (95.5%) |
| Site 6 | 16 / 17 (94.1%) | 6 / 17 (35.3%) |
| Site 7 | 9 / 19 (47.4%) | 17 / 19 (89.5%) |
| Site 8 | 6 / 20 (30.0%) | 19 / 20 (95.0%) |
| Site 9 | 4 / 6 (66.7%) | 4 / 6 (66.7%) |
| Site 10 | 5 / 7 (71.4%) | 2 / 7 (28.6%) |
| Site 11 | 2 / 4 (50.0%) | 1 / 4 (25.0%) |
| Abbreviations: n, number; OHP, obstetric healthcare professional; SD, standard deviation. | | |

Table S4. Response rate per center

Table S5. Internal consistency of NoMAD and MIDI questionnaires

|  | **T=1**  α | **T=2,**  α |
| --- | --- | --- |
| **NoMAD** | 0.80 | 0.89 |
| **MIDI** | 0.86 | 0.85 |


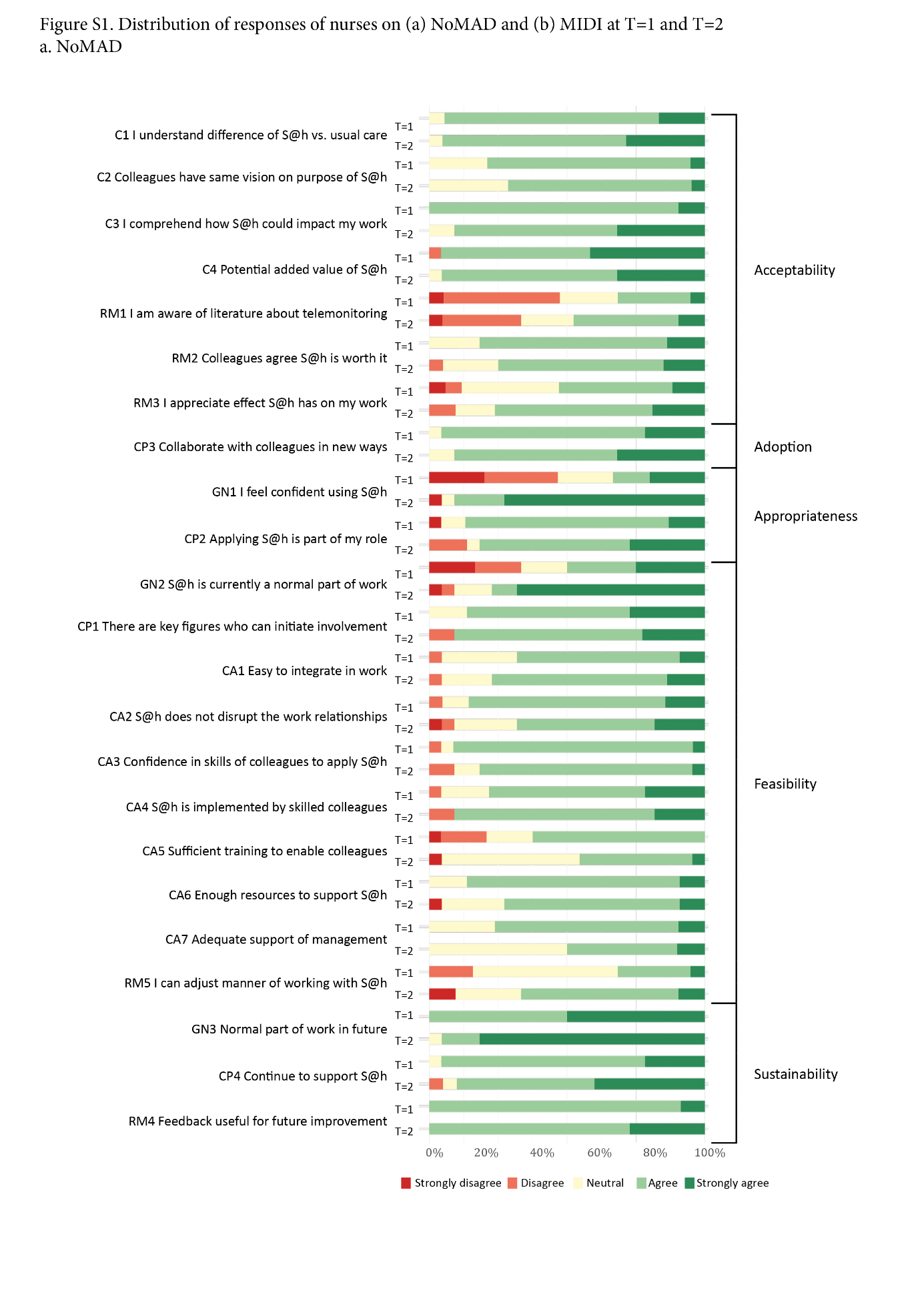


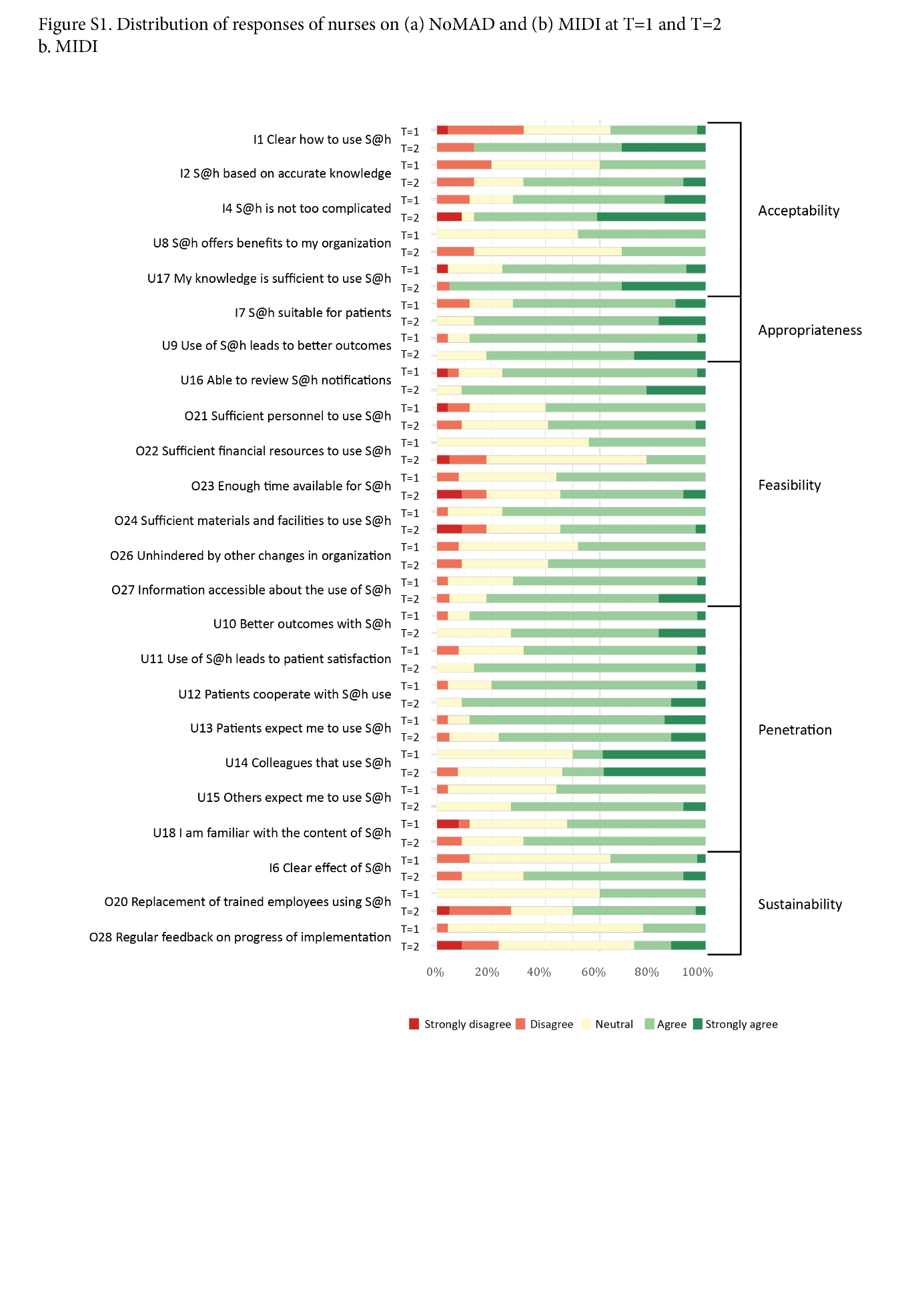

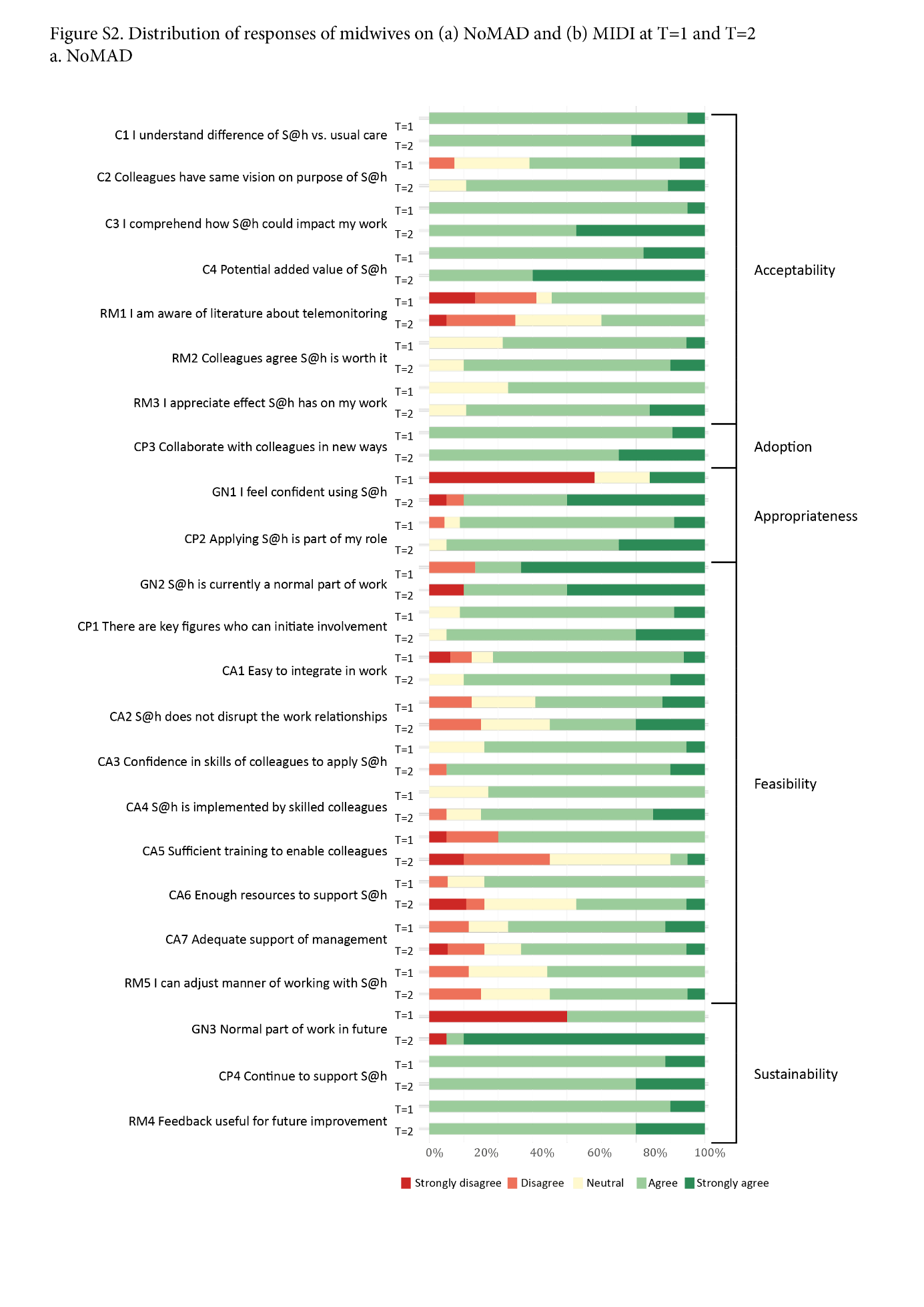

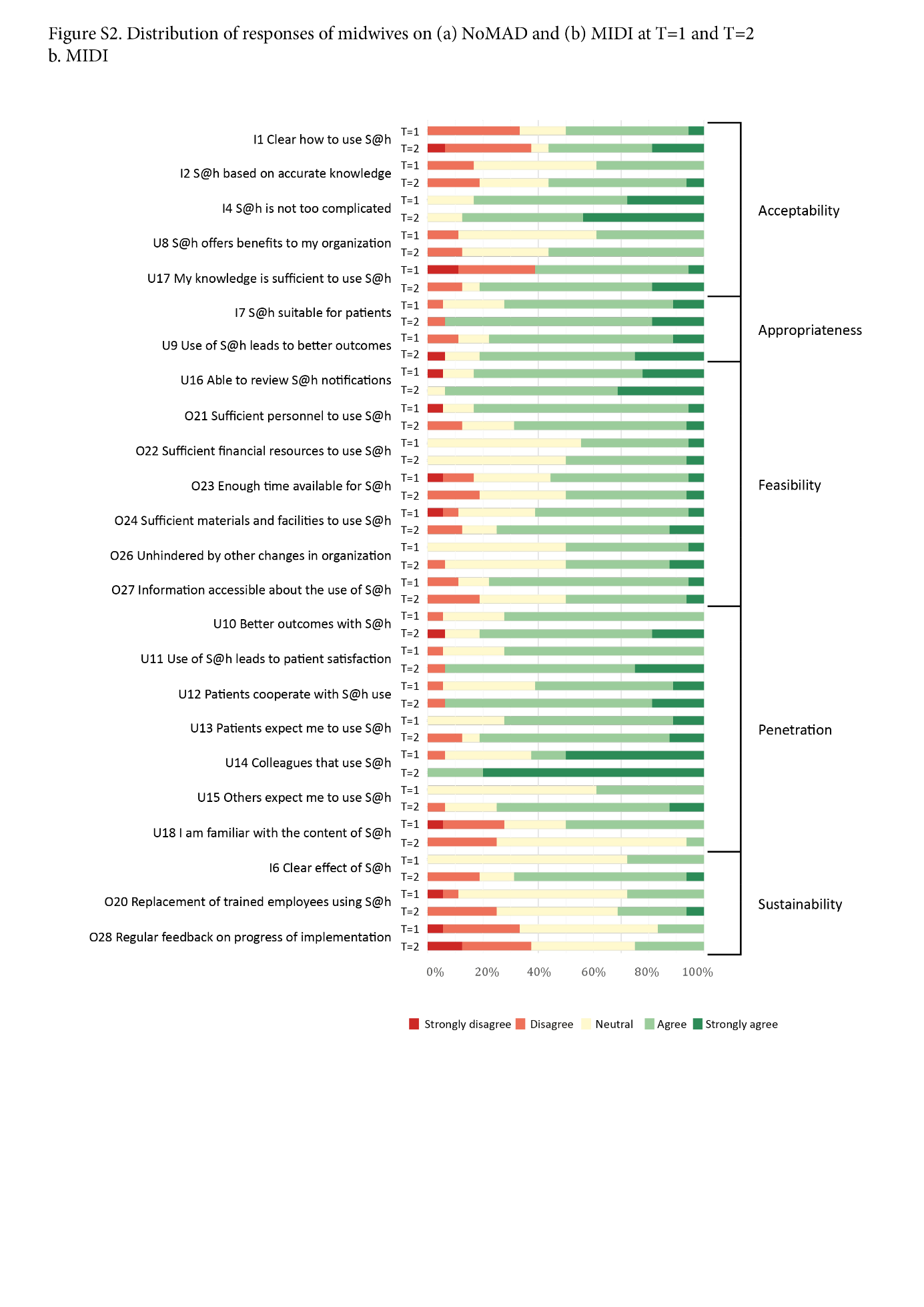


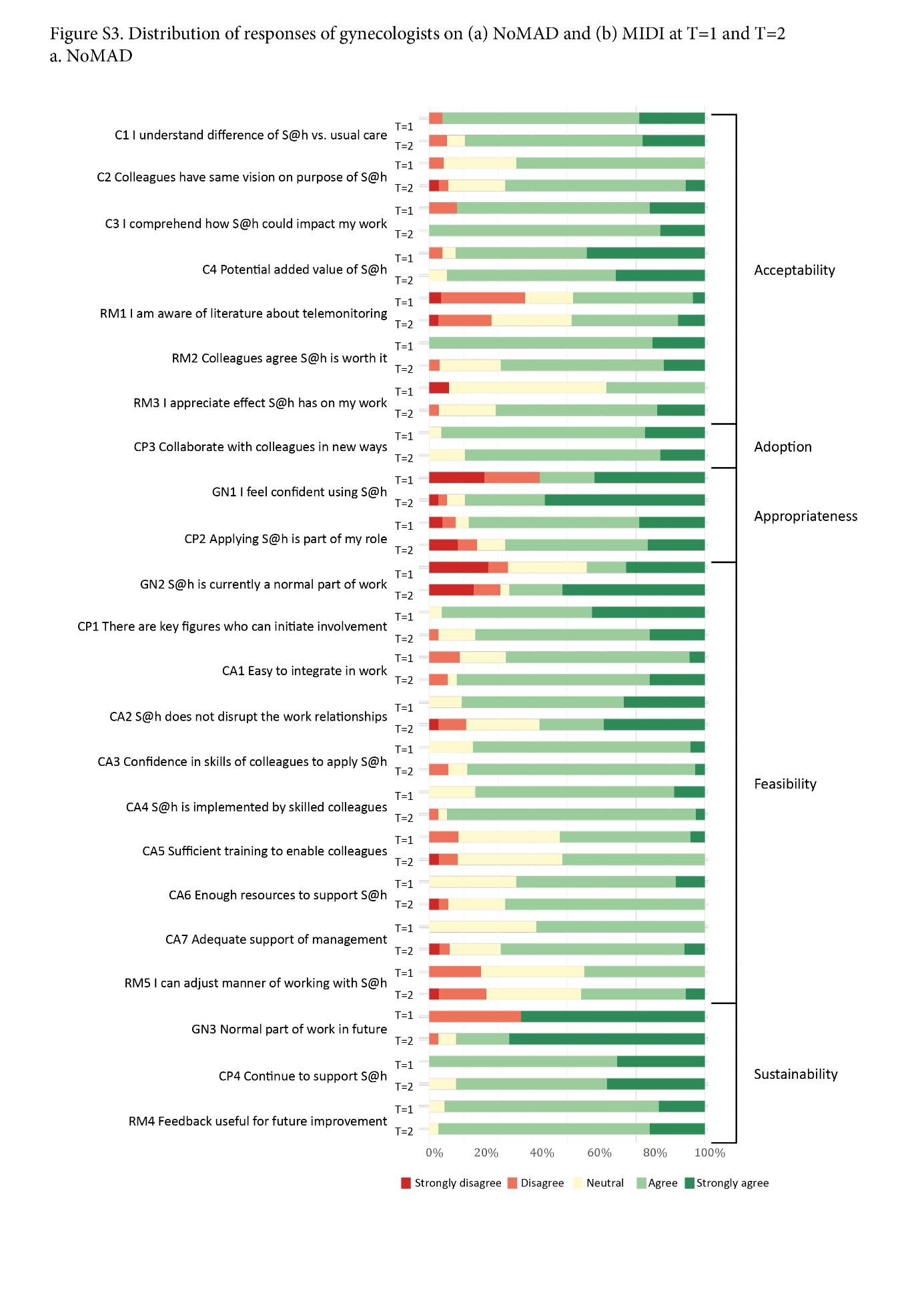

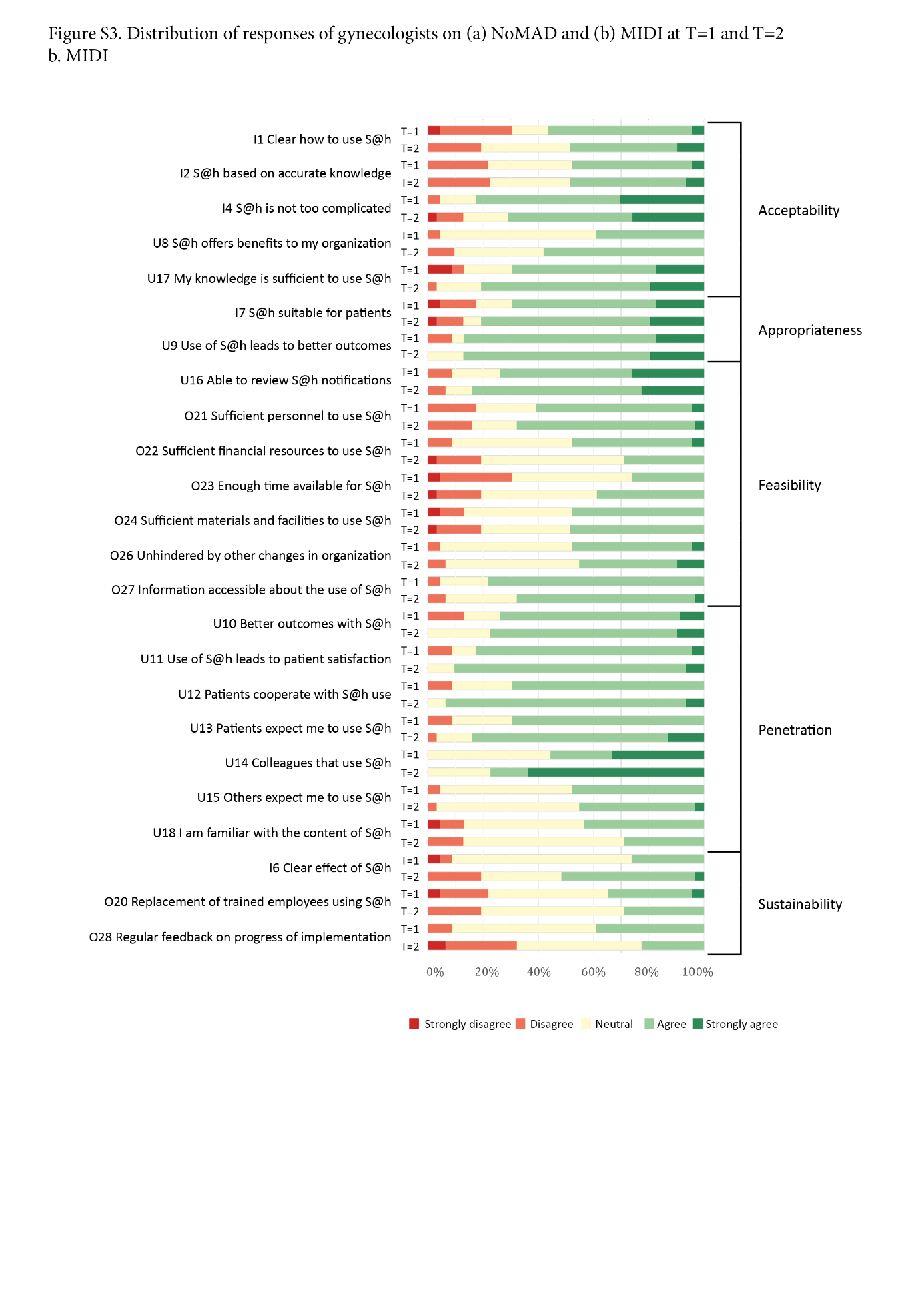

Supplement: sj-docx-1-dhj-10.1177_20552076251376518 - Supplemental material for Implementing the SAFE@home digital platform for blood pressure home monitoring for patients with (a risk of) hypertensive disorders of pregnancy: A barrier and facilitator analysis among obstetric healthcare professionals [file sj-docx-1-dhj-10.1177_20552076251376518.docx]
